# Unconventional Optical Matter of Hybrid Metal–Dielectric Nanoparticles at Interfaces

**DOI:** 10.1021/acsnano.4c10418

**Published:** 2024-11-18

**Authors:** Boris Louis, Chih-Hao Huang, Marc Melendez, Ana Sánchez-Iglesias, Jorge Olmos-Trigo, Sudipta Seth, Susana Rocha, Rafael Delgado-Buscalioni, Luis M. Liz-Marzán, Manuel I. Marqués, Hiroshi Masuhara, Johan Hofkens, Roger Bresolí-Obach

**Affiliations:** † Laboratory for Photochemistry and Spectroscopy, Division for Molecular Imaging and Photonics, Department of Chemistry, Katholieke Universiteit Leuven, Leuven 3000, Belgium; ‡ Department of Applied Chemistry and Center for Emergent Functional Matter Science, National Yang Ming Chiao Tung University, Hsinchu 300093, Taiwan; § Departamento de Física de Materiales & Condensed Matter Physics Center (IFIMAC) & Nicolás Cabrera Institute, Universidad Autónoma de Madrid, C. Francisco Tomás y Valiente, 7, 28049 Madrid, Spain; ∥ CIC biomaGUNE, Basque Research and Technology Alliance (BRTA), 20014 Donostia-San Sebastián, Spain; ⊥ Center for Materials Physics (CSIC-UPV), 20018 Donostia-San Sebastián, Spain; # Departamento de Física, Universidad de La Laguna, Apdo. 456., E-38200 San Cristóbal de La Laguna, Santa Cruz de Tenerife, Spain; ∇ Ikerbasque, Basque Foundation for Science, 48009 Bilbao, Spain; ○ CINBIO, Universidade de Vigo, Departamento de Química Física, Campus Universitario As Lagoas, 36310 Marcosende Vigo, Spain; ◆ Max Planck Institute for Polymer Research, Mainz 55128, Germany; ¶ AppLightChem, Institut Químic de Sarrià, Universitat Ramon Llull, Barcelona 08017, Spain

**Keywords:** optical Matter, optical trapping, hybrid nanoparticles, numerical calculations, single-particle tracking, optical machines, colloidal self-assembly

## Abstract

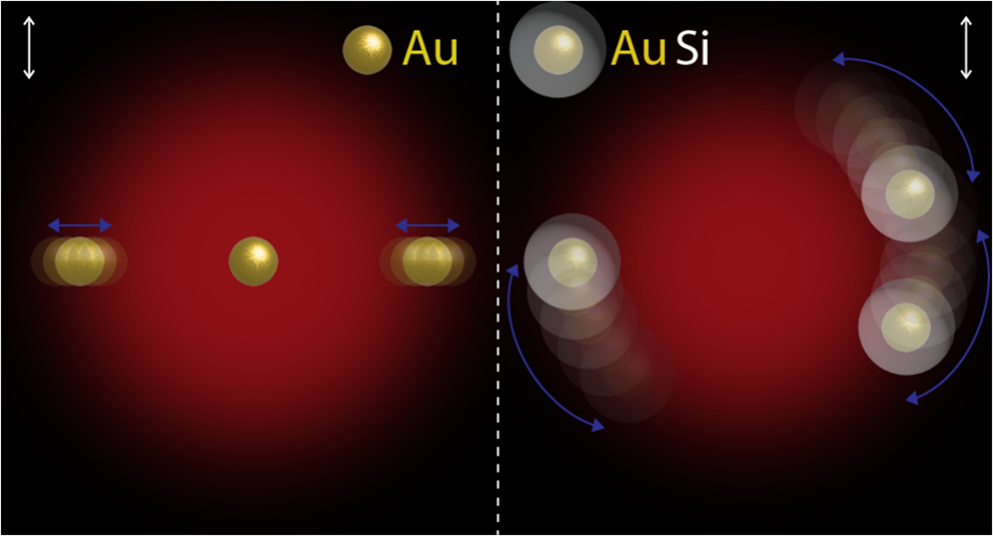

Optical matter, a transient arrangement formed by the interaction of light with micro/nanoscale objects, provides responsive and highly tunable materials that allow for controlling and manipulating light and/or matter. A combined experimental and theoretical exploration of optical matter is essential to advance our understanding of the phenomenon and potentially design applications. Most studies have focused on nanoparticles composed of a single material (either metallic or dielectric), representing two extreme regimes, one where the gradient force (dielectric) and one where the scattering force (metallic) dominates. To understand their role, it is important to investigate hybrid materials with different metallic-to-dielectric ratios. Here, we combine numerical calculations and experiments on hybrid metal–dielectric core–shell particles (200 nm gold spheres coated with silica shells with thicknesses ranging from 0 to 100 nm). We reveal how silica shell thickness critically influences the essential properties of optical binding, such as interparticle distance, reducing it below the anticipated optical binding length. Notably, for silica shells thicker than 50 nm, we observed a transition from a linear arrangement perpendicular to polarization to a hexagonal arrangement accompanied by a circular motion. Further, the dynamic swarming assembly changes from the conventional dumbbell-shaped to lobe-like morphologies. These phenomena, confirmed by both experimental observations and dynamic numerical calculations, demonstrate the complex dynamics of optical matter and underscore the potential for tuning its properties for applications.

## Introduction

Since Ashkin and colleagues’ seminal work in 1986, optical trapping has revolutionized the manipulation of micro- and nanoscale objects,^1−3^ finding applications in diverse fields like materials science, biological studies, and fundamental research.^4−8^ Hence, it is now possible to gain high spatiotemporal control of different nanosized objects (e.g., dielectric and metallic nanoparticles, semiconductor quantum dots, proteins, molecular clusters) thanks to the properties of light.^8−12^ Although stable optical trapping typically requires the gradient force to be stronger than the scattering force, this requirement is lifted when the trapping takes place at an interface. This is because the interface serves as a physical barrier, causing the scattering force to also contribute to the trapping mechanism. This enables the trapping of objects that cannot be easily trapped in solution and facilitates the simultaneous capture of larger numbers of particles. As a result, large assemblies can be formed, extending tens of micrometers outside the irradiation area for dielectric particles^9−11,13,14^ and plasmonic particles,^8,15^ but also proteins^16^ and amino acids^17^ with a potential increase in crystallization efficiency by many folds.^6,7,17,18^ The underlying mechanism for the creation of such large assemblies of micro- or nanoparticles is optical binding.

Optical binding was introduced by Burns et al. in the 1990s, demonstrating organized arrangements of polystyrene microspheres using interference fields.^19,20^ The dielectric particles showed arrangements at specific distances from one another, proportional to multiples of the laser wavelength, due to a scattering-based mechanism. Consequently, optical binding was primarily studied on microparticles due to their larger scattering and slower Brownian motion.^21−24^ More recently, optical binding has also been studied on plasmonic particles where the optical binding strength can be resonantly enhanced more than 3 orders of magnitude by the presence of local surface plasmons.^25−30^ Our recent work extends these findings, demonstrating the creation of optical matter outside the irradiation area using gold nanoparticles (AuNPs) at interfaces, with a tightly focused laser beam.^31^

Apart from the fundamental interest in light–matter interactions, the assembly of nanoparticles into ordered structures has proven to be a promising approach for creating functional materials (e.g., metamaterials) with specific optical properties, such as extremely high refractive indices (*n* > 3), negative refractive indices, and optical transparency.^32−42^ Although metamaterials are typically fabricated as static structures using lithography, there has been a recent shift toward using colloidal self-assembly, enabling reconfigurability and the construction of complex three-dimensional (3D) structures.^39,43^ In this context, optical binding emerges as a promising alternative for creating organized nanoparticle structures, where the geometry and arrangement can be finely tuned by the properties of light and materials, offering full reconfigurability.^44,45^ This reconfigurability makes optical matter assemblies active colloidal self-assemblies,^46^ and therefore, they can also serve as a model system for swarming nanorobots.^47^ Thus, controlling and generating optical matter both inside and outside the irradiation area has the potential to create larger and more specialized structures, including optical machines,^23,48^ optical crystals^45^ for applications in light manipulation, microfluidics, and microrheology.^45^

However, designing such applications requires a deeper understanding from both theoretical and experimental perspectives, necessitating an exploration of relevant parameters, such as particle properties (size, shape, and composition) and light properties (wavelength, power, and polarization). For instance, most studies focus on dielectric or metallic particles, which represent two extreme cases with specific balances of gradient and scattering forces, investigating hybrid materials could illuminate the individual contributions of these forces, enabling tuning of the force balance for specific optical matter arrangements. The behavior of hybrid metal–dielectric systems has only been studied from the perspective of single-particle optical trapping,^49^ or theoretically,^50−53^ until now. Furthermore, theoretical studies have often employed static approaches that calculate configuration energy minima, whereas optical matter is highly dynamic, and the experimentally observed structure may not represent the lowest energy state.^54,55^ Thus, dynamic calculations are needed to fully understand these material structures.

Here, we investigate hybrid submicron particles composed of a 200 nm diameter gold core coated with silica shells of different thicknesses, ranging from 0 to 100 nm. We investigate the optical binding and optical matter formed by these particles using optical trapping at an interface. We compare their arrangement and the correlation between particle motions and binding strength as a function of the silica shell thickness. Additionally, we also compare the experimental results with numerical calculations in which silica shell thickness and electrostatic radius are discussed. We show that increasing the silica shell thickness leads to a decrease in the interparticle distance below the expected optical binding length. With large silica shell thickness (>50 nm), we observed a significantly different arrangement (hexagonal as opposed to oriented perpendicular to the polarization) as well as rotative oscillations despite the absence of light-induced angular momentum. The difference in arrangement and rotative oscillations is also confirmed by our calculations of particle dynamics based on multiple scattering optical binding, hydrodynamic interactions, and electrostatic forces. In addition to these individual-level studies, we found the dynamic swarming morphology of many NPs changes from dumbbell-shaped to lobe-like due to the silica layer. This effect could be suppressed using a solvent to match the refractive index of the silica layer. Our results deepen the understanding of the formation and control of optical matter from a theoretical and experimental point of view, which is crucial for the rational design of periodical optical matter structures.

## Results

### Microscopy Observations of Particle Assembling Into Optical Matter inside the Irradiation Area

Throughout this work, we used a home-built widefield system with an air objective (Olympus, 0.9 NA), which was used to both (1) focus a 1064 nm Nd:YAG laser (linearly polarized along the *y*-axis) at the water–glass interface of the sample and (2) image the forward scattering of the nanoparticles using a dark-field condenser. The hybrid metal–dielectric particles were then trapped and observed at the interface around 1.5 μm below the focal point of the laser.

**Figure 1:**
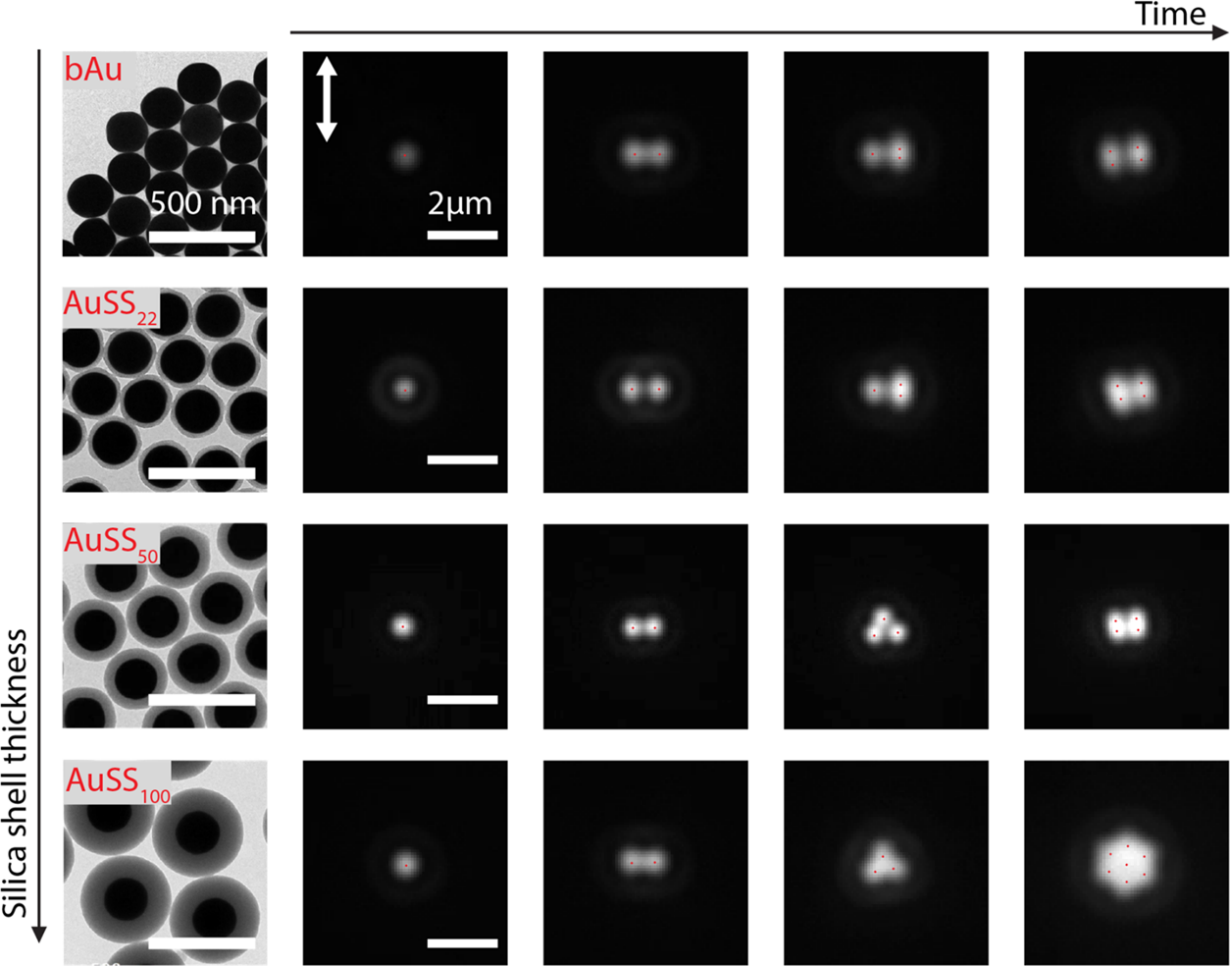
Scattering images showing the initial assembly process of AuNPs with different silica shell thicknesses. The diameter of the gold core is 200 nm, while the silica shell varies from 0 to 100 nm. The white arrows indicate the direction of the linear polarization of the trapping laser. The red dots depict approximated particle positions, guiding the reader to see the different conformations.

First, we looked at the gradual formation of optical matter during optical trapping of hybrid gold nanoparticles (AuNPs) with silica shells (SS) of differing thicknesses ranging from 0 to 100 nm at the water/glass interface. Notably, the size of the Au core is the same for all of the particles. Figure 1 shows the results for silica shell thickness of 0, 22, 50, and 100 nm. The different samples will be termed bAu (bare Au), AuSS_22_, AuSS_50_, and AuSS_100_ NPs; representative transmission electron microscopy (TEM) images of the silica-coated gold nanoparticles are shown in Figure 1.

In every instance, the first NP arrives at the center of the laser focus approximately 1 min after switching on the trapping laser (1064 nm, 20 mW, see Figure 1). Typically, the second NP aligns in the direction perpendicular to the laser polarization, with the laser focus located at the midpoint between both particles. However, in about 10–20% of cases, parallel alignment with respect to the laser polarization was observed, most probably due to enhanced near-field interactions. The orientation in the two-particle case and the near-field interactions are explained in more detail in the section “Two particles case, experiment vs theory.” Note that perpendicular alignment is consistent with the dipolar nature of scattering by bare 200 nm AuNPs, as previously reported.^8,31,56^ In the case of bAu, AuSS_22_, and AuSS_50_ NPs, when additional particles are introduced, they tend to form a rectangular pattern with the long axis aligned perpendicularly to the laser polarization due to a combination of the near-field (parallel alignment) and the far-field contributions together with an additional short-range repulsion (perpendicular alignment) effect. Conversely, AuSS_100_ NPs exhibit a hexagonal pattern. Of note, AuSS_50_ NPs occasionally display behavior that falls between that of bAu and AuSS_100_ NPs, as can be seen for the case of 3 trapped NPs, which present a triangular conformation similar to AuSS_100_. To understand the differences qualitatively shown in the first section, we examine the particle dynamics of the initial stage in more detail. By using a highly diluted dispersion, the number of particles inside the optical trap was kept low, which enabled us to calculate their position with high accuracy and reconstruct their spatial trajectory by single particle tracking. In addition, to understand the role played by each interaction, we performed numerical simulations of the particle dynamics considering optical forces (including the primary field and multiple scattering), steric and electrostatic forces, as well as hydrodynamic interactions. Simulations carefully consider the experimental conditions for the incident light (optical axis and polarization), trapping near a horizontal glass–water interface, and the optical response of the hybrid metal–dielectric nanoparticles (see the Materials and Methods section and Figure S1 for further details). In the following sections, we will focus on the particle motion when only 2 or 3 particles are trapped from both the experimental and theoretical points of view. Note that the theoretical data points are always presented with “open symbols” while the experimental data points are presented using filled symbols.

### Two-Particle Case, Experiment Vs Theory

**Figure 2:**
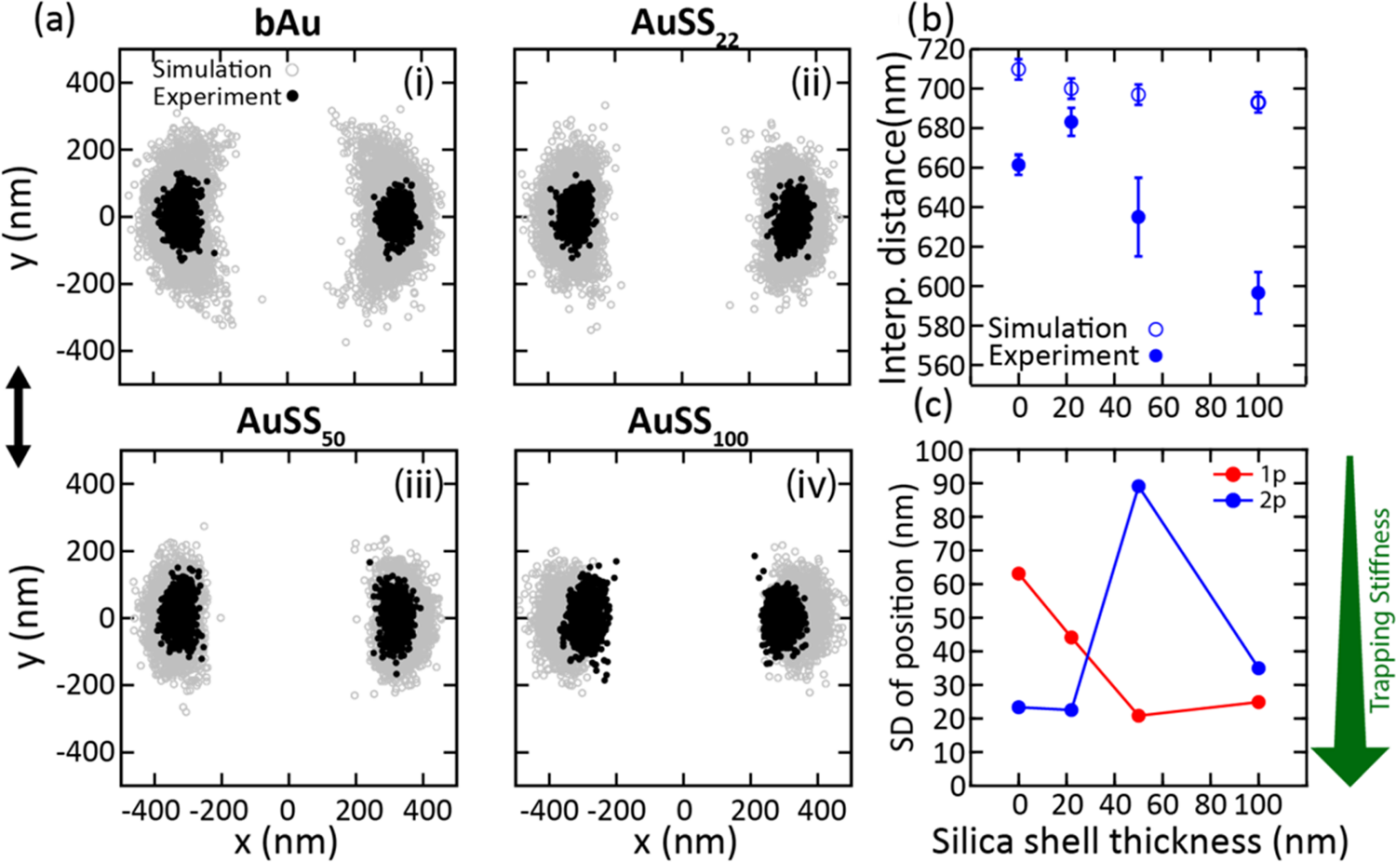
Single-particle tracking analysis of two optically trapped AuNPs coated with silica shells of different thicknesses. (a) Comparison between experimentally representative localization maps (black dots) and numerical calculations (gray dots) for the four values of silica shell thickness tested: (i) bAu NPs, (ii) AuSS_22_ NPs, (iii) AuSS_50_ NPs, and (iv) AuSS_100_ NPs. (b) Experimental and simulated interparticle distances (center-to-center) as a function of silica shell thickness. Error bars indicate the average standard deviation of the interparticle distance across 10 different experiments. (c) Relative binding strength against silica shell thickness, determined by comparing the standard deviation(s) of the motion for a single particle and for two particles. As a visual aid, the green arrow indicates the growing trapping stiffness.

Experimental and numerical calculations for systems with two particles are presented in Figure 2. Both experimental data and numerical calculations show optical binding for the two-particle system, with particles oriented in the *x*-direction, i.e., perpendicular to the trapping light polarization. The nanoparticles occupy symmetrical positions on the *x*-axis, separated by around 600–700 nm. More experimental examples are provided in Figure S2. We note here that this distance is smaller than the expected distance for optical binding, which should be closer to the laser wavelength in the medium (λ_trapping_/*n*_medium_ ∼ 800 nm), indicating that other forces also contribute to this condition.^19,20^

The alignment and center-to-center distance between the two NPs are governed by a complex force balance that involves optical forces (primary optical gradient and scattering), alongside short-ranged repulsive electrostatic forces. Of note, the electrostatic forces cannot be ignored because the employed NPs have a relatively large negative ζ-potential (i.e., indicative of the surface charge), ranging from −25 to −31 mV. As depicted in Figure 2b, an increase in silica shell thickness correlates with a reduction in interparticle distance. Numerical calculations (Figure S3) show that the effect of optical forces is to drive the particles to a symmetrical sticking position along the *y*-axis (parallel to the laser polarization), corresponding to the near-field configuration observed in the experiment (10–20% of cases), in which the NPs are much closer than the trapping wavelength in the medium. Only by introducing the short-range repulsion do the particles move to positions along the x-axis (perpendicular to the polarization), with a separation distance of about 650 nm, similar to the value found in experiments (660 to 590 nm for bAu to AuSS_100_, see Figure 2). The importance of the electrostatic repulsion becomes evident when plotting the isolated effect of the optical forces. In Figure S3, we plot the optical force versus the particle-focus distance for two particles at *y* = 0, symmetrically placed around the focus along the *x*-direction. Notably, for a bare AuNP (no shell), the optical force is attractive for every position, and there is no equilibrium point. Adding an extra repulsion is needed to obtain a stable equilibrium distance where the force is zero and restored (negative x-slope). Interestingly, simulations show that, in particles with a silica shell, equilibrium configurations can be induced just by adding the shell-induced modification of the particle polarizability (i.e., even without electrostatic repulsion).^57^ In this case, the equilibrium distance increases with the shell thickness. However, the trend observed in the experiments is the opposite. To understand this effect, we note that growing the silica shell increases the effective electrostatic radius of the particle (i.e., the particle radius plus Debye length), leading to a reduction in its surface charge density and less particle–particle electrostatic repulsion.^58^ Adding electrostatics to the simulations confirms this trend, albeit to a lesser extent than that in experiments. Capturing these fine details in simulations requires careful tuning of electrostatics and probably also considering minor contributions (e.g., thermophoresis), which we have likely oversimplified.

To further analyze the particle dynamics, we have looked at the Pearson correlation coefficient between the time-dependent position of the two particles and compared the correlation for the *x*-axis (perpendicular to the polarization) and the correlation in angle (see Figure S4). bAu NPs and AuSS_22_ NPs demonstrate highly correlated motion along the *x*-direction, perpendicular to laser polarization, which is characteristic of optically bound particles by a pure dipole metallic scattering mode. Notably, AuSS_50_ and AuSS_100_ NPs exhibit rotative oscillations around the laser focus, as shown in Figure S4, where the interparticle correlation of motion in the *x*-direction is compared with the angle derived from a polar coordinate transformation. An unexpectedly high angular correlation is observed for both AuSS_50_ and AuSS_100_ NPs. This is surprising because the trapping laser is linearly polarized and, therefore, it does not transfer any net angular momentum to the particles through light–matter interactions. As confirmed by simulations, this apparent rotation is due to a thermally induced drift around an optically stable configuration.

The force field that traps the particle around the center of the optical trap can be regarded as a harmonic force field. Following Boltzmann’s statistical theory, the trapping stiffness of an optical potential well is inversely proportional to the square of the standard deviation(s) (SDs) of the position of the particle(s) due to thermal fluctuations.
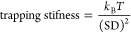
Therefore, measuring the SDs for the particles is a direct way to measure the trapping stiffness. Figure 2c shows the SDs for one- and two-particle configurations as a function of silica shell thickness. Considering only the trapping force, we expect one particle to be more tightly trapped than two, since the single particle is placed exactly at the focus, whereas the two-particle system must be positioned symmetrically around the focus. Moreover, from Spadaro et al.,^49^ we expect the trapping stiffness to increase (i.e., standard deviation decreases) with the dielectric SiO_2_ shell thickness. However, from the comparison of one- and two-particle systems (Figure 2C), it turns out that pure gold was up to 3.5 times more strongly trapped in the two-particle case compared to the single-particle case. This confirms that pure metallic particle-induced optical binding is a strong optical force that coheres the generated optical matter. On the other hand, a thick silica shell results in destabilization, meaning that the stiffness for two optically bound silica-coated particles is actually lower than when a single silica-coated particle is present in the trap. It should be noted that, for the single-particle case, the stiffness still increases (standard deviation decreases) with the silica shell thickness, which is in agreement with Spadaro et al.^49^ The effect observed in Figure 2c is thus due to the two-particle case being significantly more stabilized at low shell thickness compared to that at large shell thicknesses. The trapping stiffness in the two-particle system results from balancing the optical binding, optical scattering, optical gradient, and electrostatic repulsion forces. Notably, the two AuSS_100_ NPs are more stiffly trapped compared to the AuSS_50_ case. This experimental evidence confirms that the larger dielectric part of the former leads to significantly stronger gradient forces, allowing them to overcome the weaker optical binding and electrostatic forces. Indeed, this force balance supports the difference in the optical trapping behavior of AuSS_100_ NPs. Finally, it should be mentioned that the apparent resolution of our tracking under these measurement conditions is around 20 nm. As a consequence, Figure 2c does not show any data point below 20 nm, which also determines the limit of detection in trapping stiffness.

### Three-Particle Case, Experiment vs Theory

**Figure 3:**
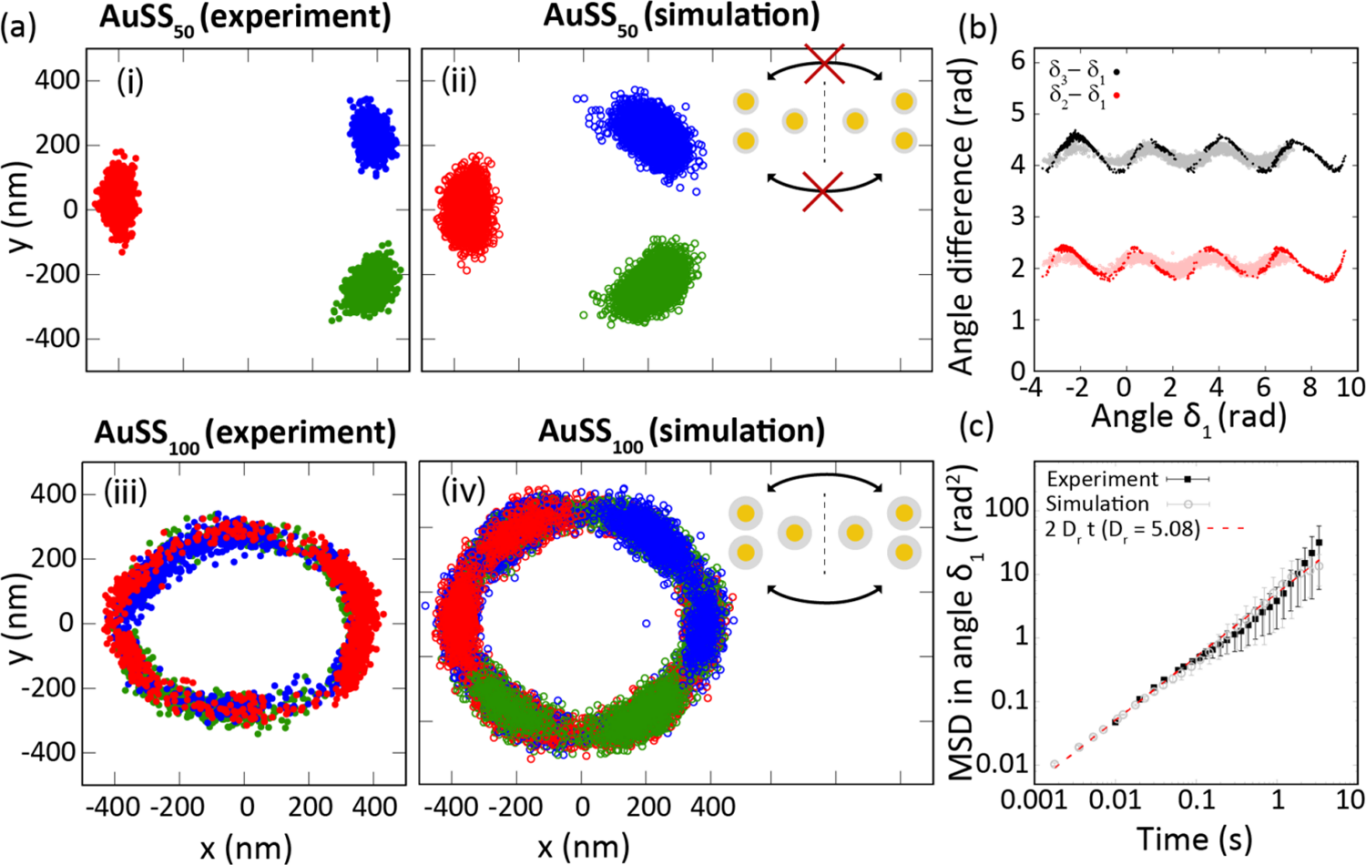
(a) Representative localization maps of three optically trapped AuNPs for (i) AuSS_50_ exp., (ii) AuSS_50_ Sim., (iii) AuSS_100_ exp., and (iv) AuSS_100_ Sim. As a visual aid, a scheme of the two configuration states is included in panels (iii) and (iv). (b) Angle difference versus orientation angle of a reference particle for a triangle of three trapped hybrid metal–dielectric 200 nm diameter gold spheres with a silica shell of 100 nm. In the simulations, the temperature was set to 40 °C, and the electric double layer exclusion distance was set to 110 nm. Dark dots correspond to experimental data, whereas the light color represents numerical values. (c) Mean square displacement (MSD) of the angle δ_1_ versus time for experimental and simulated systems.

Inspired by the incipient rotative oscillations observed for two trapped particles, we investigated the effect of adding a third particle to the system. Figure 3a presents localization maps for cases with three trapped NPs, with a focus on AuSS_50_ and AuSS_100_ for both experiment (left) and numerical calculations (right). Additionally, Figure S5 displays experimental examples for all four silica shell thicknesses. Typically, the third NP positions itself adjacent to one of the previously trapped NPs, forming an isosceles triangle arrangement. The distance from the two NPs on the shorter side of the triangle to the opposite vertex (here along the *x*-axis) remains close to the trapping light’s effective wavelength due to optical binding. In contrast, the distance between the closer particles increases with the silica shell thickness.

Similar to the two-NP system, a thicker silica shell was found to induce a decrease in the level of optical binding, leading to increased rotational oscillations in NP assemblies and even complete rotations for larger silica shells. For instance, the distribution of AuSS_50_ NPs exhibits elongation along a circular path (see Figure 3a–iii). As a general trend, numerical calculations reproduce the experimental measurements and unveil the origin of the rotation pattern. The three particles can arrange themselves into two configurations: the triangular shape shown in Figure 3a or its mirror image with respect to the *y*-axis. However, due to the high rearrangement activation energy, they cannot thermally rearrange between these two configurations within the observation time scale. Instead, the picture changes for the AuSS_100_ NPs. As shown in Figure 3, for a 100 nm dielectric layer, the particles start to move circularly around the focus with no preferential rotational direction. However, a detailed frame-by-frame analysis reveals that the three NPs still arrange themselves into two metastable configurations, similar to those observed for AuSS_50_ NPs (see Figure 3a–ii inset). Thus, the rotation arises from a coordinated and thermally activated transition between these two metastable configurations, which have a lower rearrangement activation energy or receive more energy due to their higher volume, subjecting it to stronger light–matter interaction (i.e., more photons; see Figure 3a–iv inset). Additionally, for AuSS_100_ NPs, the movement of the small assembly follows a circular pattern even under linear laser polarization. Owing to its thermal (random) origin, the direction of the circular motion is stochastic and can vary during observation, likely resulting in an average rotation of 0 radians. Notably, this circular motion was only observed for particles with shells thicker than 50 nm. Using numerical simulation, the circulation for small particles was absent when the electrostatic exclusion layer was decreased to 80 nm. This detail confirms, again, the crucial role played by electrostatic interactions in the configurations adopted by the trapped NPs.

To quantitatively analyze this cooperative circular motion, we tracked the position vector of the particles with respect to the geometrical center of the three NPs and the angle formed between this vector and the *x*-direction. Figure 3b shows the dynamic phase space by plotting the angle difference between particle “*I*” and particle 1 (for *I* = 2, 3) against the angle of particle 1 (see the geometrical representation in Figure S6). If the triangular arrangement is highly rigid, then the angle difference should not change. Both experiments and simulations show that the particles do not move circularly as a rigid body, i.e., the angle difference does not remain constant. As the “optical matter” collectively transits from one metastable configuration to the other, the angle between the particles varies because it depends on the orientation of the entire triangular arrangement. Note that simulations are in exceptionally good agreement with experiments yet leading to a slightly softer triangle deformation (smaller amplitudes). These calculations results were achieved by slightly increasing the temperature from 20 to 40 °C and the electrostatic repulsion layer from 100 to 110 nm. As shown in previous studies, an increase of roughly 20 °C in the temperature of the surrounding water due to the laser heating the particles is realistic^59^ and would cause an increase in the repulsive electrostatic double layer because the Debye length grows with temperature.^60^ Even after careful adjustment of these parameters to better mimic the experimental conditions, the accurate amplitude of fluctuations in the angle is not fully captured by the simulations. The experimental system constrains the lateral motion of the NPs more tightly than the simulations. Again, this might be due to the simple (pairwise) repulsive force approach that we are considering. Nevertheless, by using the angular mean squared displacement (MSD) (see Figure 3c), we show that the speed of the circular motion accurately reflects the experiments.

### Optical Matter in the Photostationary State

So far, we have looked in detail at the formation of optical matter at low particle numbers (1–3) and already observed striking differences at different silica shell thickness. As the number of particles increases, the system becomes increasingly complicated and the number of possible arrangements increases exponentially. Since it would be impossible to describe them all, here we look at the photostationary state, which is reached when the number of particles in the optical matter remains approximately constant.

**Figure 4:**
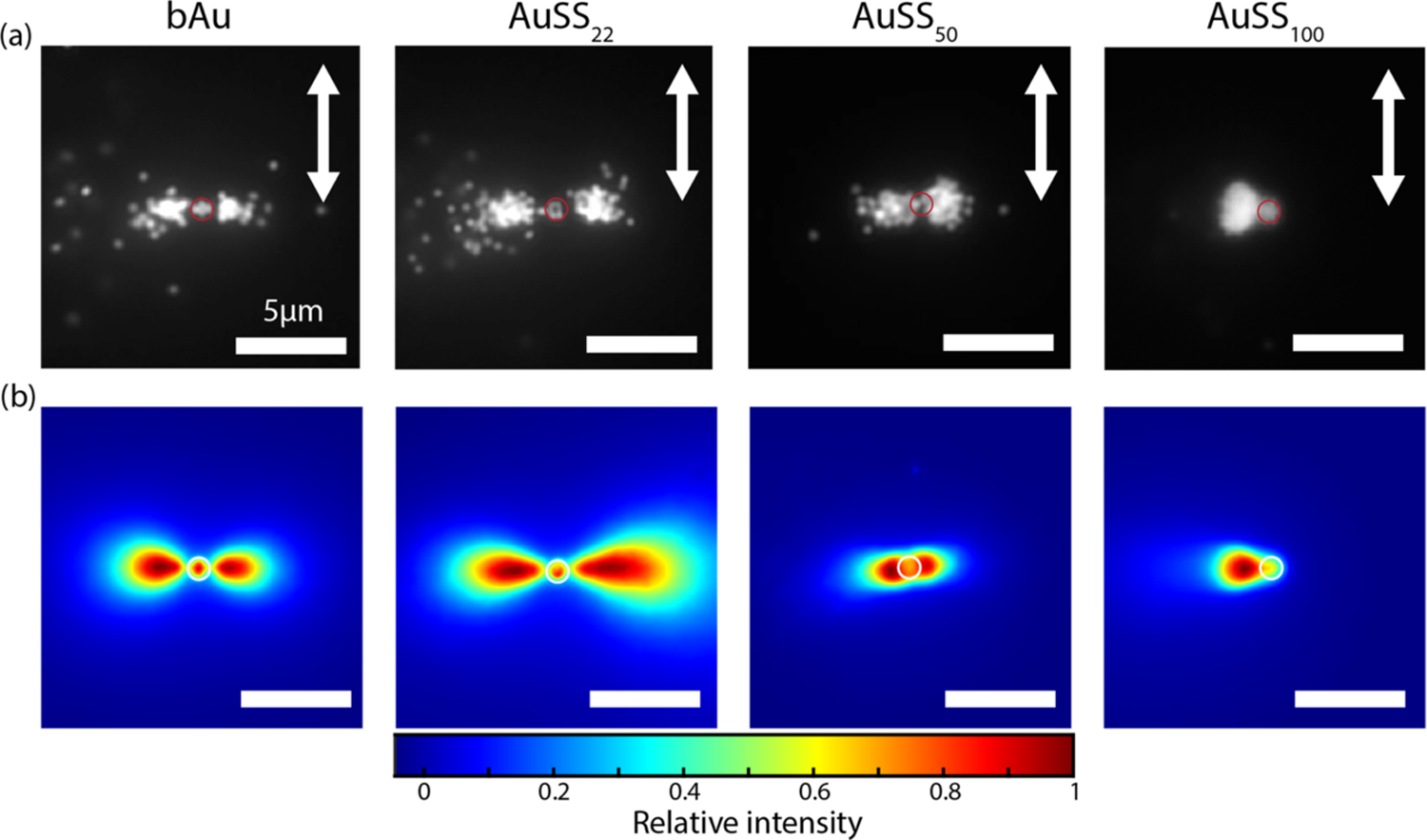
Scattering images showing Au NP assemblies upon laser irradiation. (a) Exemplary frame of steady-state assembly for different silica shell thicknesses: bAu, AuSS_22_, AuSS_50_, and AuSS_100_ NPs. (b) Time-averaged intensity, which corresponds to the probability density function (PDF). The white arrows indicate the direction of linear polarization of the trapping laser. The red and white circles approximately depict the laser focus position and size. The length of scale bars are 5 μm.

Figure 4a displays scattering images for all samples (bAu NPs, AuSS_22_ NPs, AuSS_50_ NPs, AuSS_100_ NPs) in the photostationary state (>10 min irradiation, particle number approximately constant). Across all samples, the particle assemblies are observed to be significantly larger than the focal spot, which is consistent with previous findings.^8,10,31^ For bAu NPs and AuSS_22_ NPs, the assembly shape resembles a dumbbell, similar to the previously reported swarming behavior of bare gold particles,^8^ as expected if the only significant optical force contribution arises from a pure metallic dipolar scattering mode. The assembly of AuSS_50_ NPs also displays a dumbbell shape in most cases (shown in Figure 4a). However, a variety of shapes were also observed (Figure S7). In contrast, AuSS _100_ NPs form asymmetric assemblies comprising a large lobe with a high density of particles on either side of the laser focus. Indeed, releasing and retrapping the particles (laser on and off) can result in changing the large lobe position (i.e., right or left of the focus). Notably, the orientation of these assemblies can be manipulated by altering the polarization direction, as shown in Figure S8.

Due to the high number of particles with overlapping signals, particle tracking cannot be applied successfully here. As an alternative to particle tracking, since intensity relates directly to the number of particles, computing the time-average intensity of the steady-state assembly will give us a map that directly correlates with the probability density function (PDF) or the probability of finding a particle at a specific position. Figure 4b shows the normalized time-averaged intensity of the steady-state assembly for all samples. We can clearly observe how the shape varies for different silica shell thicknesses. From the approximated PDF, we can see that bAu and AuSS_22_ have clear dumbbell shapes, whereas AuSS_50_ presents a much more contracted version of the dumbbell, and AuSS_100_ has completely lost the dumbbell characteristic. Instead, it presents a small lobe, likely reflecting the hexagonal arrangement inside the irradiated area as observed at a smaller number of particles (Figure 1), and a large lobe completely outside the irradiation area, positioned at the left or the right of the laser focus.

**Figure 5:**
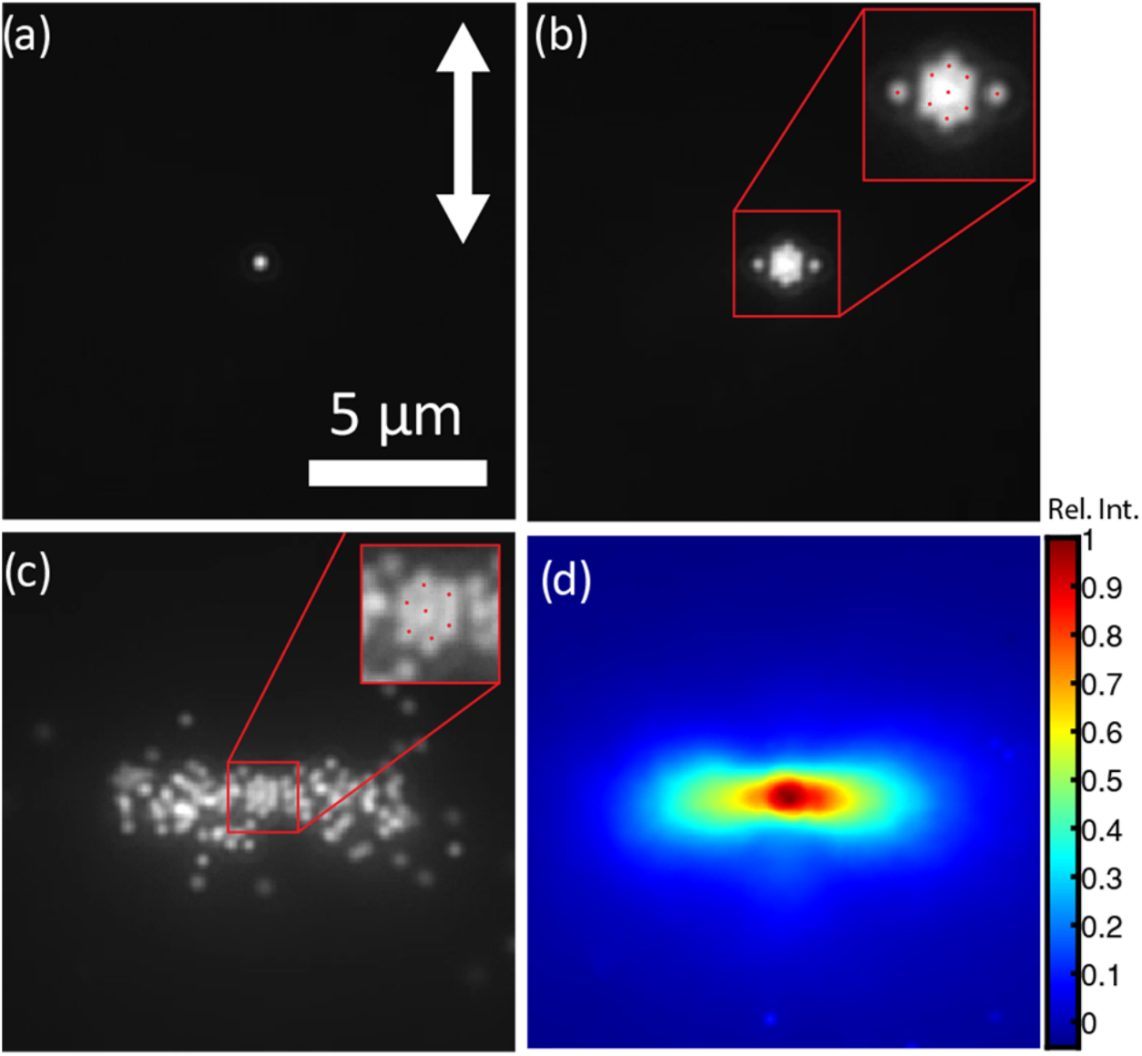
Effect of refractive index on the optical matter for AuSS_100_ NPs. (a–c) Sequential scattering images showing the assembly evolution from (a) 1 particle, (b) hexagonal arrangement, (c) to full assembly when the sample solvent is changed from water (*n* = 1.33) to DMF (*n* = 1.43). Note that DMF almost perfectly matches the refractive index of the Silica shell. The white arrows indicate the direction of linear polarization of the trapping laser. The scale bars refer to 5 μm.

To elucidate the effect of the silica shell on optical matter formation, we replicated the assembly of AuSS_100_ NPs in *N*,*N*-dimethylformamide (DMF), a solvent that matches the refractive index of the silica shell, thereby making the shell nearly optically transparent. More specifically, neither reflection nor refraction occurs at the interface between silica and DMF. Figure 5 shows the optical matter formation at different stages from a single particle (a) and hexagonal arrangement (b) to the full assembly (c). When DMF was used as a refractive index-matching solvent, the dumbbell-shaped assembly was partly restored (Figure 5c). Despite the solvent change, the initial assembly stages (Figure 5b) are quite similar to those observed in water, forming a hexagonal, instead of a linear, arrangement inside the irradiated area with the ability to form optical bonds outside the irradiated area (see the two nanoparticles trapped at the right and left sides of the central alignment). However, as the number of particles increases, the assembly expands symmetrically on both sides, resulting in the photostationary state being very similar to the bare gold case except for the central hexagonal arrangement. Figure 5d shows the normalized time-averaged intensity of the steady-state assembly, this time showing a clear symmetric dumbbell shape. The central part is however broader and more extended in the y direction due to the hexagonal arrangement, which is still maintained in the optical matter despite the refractive index change. This is a clear difference from the case of bare gold in water, where particles orient perpendicular to the polarization and do not form a hexagonal arrangement. Finally, we note that the bare gold case in water (Figure 4b) presents some “gap” between the central alignment and the dumbbell’s side lobes, where the probability of finding a particle is very low. This is also not recovered via refractive index matching, as shown in Figure 5d.

## Discussion

To understand the observed differences in behavior, it is crucial to acknowledge that the particles under investigation consist of a gold core exhibiting plasmonic properties, surrounded by a silica shell, characterized by dielectric properties. The ratio of plasmonic to dielectric components varies among the particles. According to Figure S10, we calculated these ratios, finding dielectric volume ratios of 0, 46, 70, and 88% for bAu, AuSS_22_, AuSS_50_, and AuSS_100_ NPs, respectively. The surface ratio yields corresponding values of 0, 35, 55, and 70%. The rest of the discussion will be addressed, keeping these values in mind.

At a low particle number, for particles with the largest shell thickness (100 nm), a hexagonal arrangement predominates over the optical binding seen for smaller sizes, a likely result of the gradient force from the dielectric nature of the shell. This is corroborated by the 88% dielectric composition of the thickest shells. An increase in silica shell thickness not only improves trapping and assembly but also suggests a stronger optical force, possibly due to enhanced gradient and scattering forces. Whereas optical forces are overall stronger for the bigger particles, the optical bond is comparatively weak when compared to the single particle tweezing, as shown by the ratio between the trapping stiffness of single-particle and two-particle cases (Figure 2c). Finally, circular correlation starts to be observed at high dielectric volume ratios.

The simulations presented above model short-range electrostatic repulsion by means of a simplified Weeks–Chandler–Andersen (WCA) layer (Materials and Methods below). As already mentioned, we may consider more elaborate approaches for the short-range interaction, as in the Gouy–Chapman theory, predicting an electrostatic interaction potential between two suspended spheres of radius *R* equal to
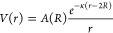
In this equation, κ stands for the inverse of the Debye length (equal to about one micron in pure water), *r* stands for the center-to-center distance between spheres, and *A* stands for a constant proportional to the zeta potential of the spheres, decreasing with the sphere radius and given by^60^
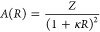
with *Z* being a constant used to tune the intensity of the potential. Gold and silica particles typically have negative zeta potentials on the order of a few tens of millivolts, so rough estimates for the constant *A* lie in the range of 100 to 1000 in simulation units (*k*_B_ × (300 K) × (200 nm)).

This repulsion might explain why experiments show a point of equilibrium for bare gold spheres not appearing in the optical forces (Figure S3). Figure S9 shows the total force, consisting of the optical interaction plus an electric double layer (EDL) potential, versus the distance for gold nanoparticles. Note that the mutual interaction between the EDL of the nanoparticles justifies the existence of an equilibrium distance like the one reported in the experiments. Also, in accordance with the experiments, this description of the repulsive force based on the double layer reverses the previous trend for the equilibrium distance versus silica thickness. Now, smaller distances are found for particles with a thicker layer, ranging from 712 nm (for bare gold) to 696 nm (for 100 nm silica). Reproducing the experimental data more precisely requires detailed knowledge of the experimental conditions of the microscopic system, beyond the scope of the current work, which is centered principally on the description of optical forces.

In fact, optical binding forces are the key ingredient to obtain the complex equilibrium configurations reported here. To probe this statement, we artificially omitted the contribution of scattered radiation in the simulations, leading to an optical force field arising solely from the external beam, and confirmed that the equilibrium configurations previously reported for two and three particles disappear. Indeed, only one particle is trapped at the focus, while all other particles diffuse far apart. On the other hand, hydrodynamic interactions do not seem to play a prominent role when a small number of nanoparticles are considered because similar equilibrium configurations are obtained when this interaction is artificially turned off.

Finally, we stress that the pairwise approximation for the EDL potential used above would not be valid for the simulations with three or more trapped particles. Even in the case of only three particles, the electric interparticle repulsive forces in the many-body case turn out to be lower than the pairwise prediction.^61^ A detailed description of the electric problem, which considers the distribution of suspended ions, hydrodynamic motion, and optical forces, would take us too far afield without contributing significantly to the conclusions of our work.

The observed circular correlation appears to be characteristic of hybrid core–shell particles and occurs only at high dielectric ratios. Indeed, such a motion derived from linear polarization has not been observed for either bare gold or dielectric particles.^10^ We hypothesize that the origin of this circular motion cannot arise through an angular momentum transfer from the trapping laser because a linearly polarized configuration is used. This motion may be attributed to a dynamic equilibrium between mirrored stable conformations, as seen with AuSS_100_, supported by observed stochastic changes in a circular direction (Figure S5b). Simulations indicate that thermal fluctuations cause alternation among these conformations. For shells of 0, 22, and 50 nm, optical forces generate strong restoring torques against rotation, whereas spheres with the thickest shells experience minimal torques, thereby facilitating easy transitions between configurations (Figure S11).

When we look at the assembly level, particles with low dielectric volume ratios (bAu and AuSS_22_ NPs) mimic the behavior of bare AuNPs, forming dumbbell-shaped optical matter. Conversely, particles with high dielectric volume ratios (AuSS_100_) exhibit a behavior distinct from that of both bare gold and pure dielectric particles. Intermediate ratios (AuSS_50_) show unpredictable behavior, indicating a transitional phase between the different morphologies.

To highlight the effect of the silica shell, we made a control experiment in which we matched the solvent to the refractive index of silica, making it effectively optically transparent. This change reinstated the swarming assembly structure for AuSS_100_ NPs, underscoring the influence of dielectric properties on particle dynamics. However, with fewer particles, the behavior more closely resembled hexagonal packing in water, suggesting that surface charge plays a significant role at low particle counts, a conclusion supported by our numerical analyses.

The details of the many-particle configurations constitute an intractable problem from an analytical point of view. It also lies beyond our simulation capacities, with the current implementation of our numerical algorithms. Nevertheless, simulations suggest that the structures observed form by first trapping a few (less than ten) particles within the focal spot. Subsequent particles arrive and lie outside but form optical bonds with the particles inside the spot (Figure 5). The longer bonds that form perpendicular to the direction of polarization exhibit greater stability, so elongated structures tend to form along that perpendicular direction. Our numerical results hint at the possibility that the asymmetry observed with AuSS_100_ NPs might arise due to asymmetric configurations with respect to the polarization that forms within the focal spot for these NPs. Moreover, the large lobes emerge as the result of complex many-body interactions with the optical force field created by the interference pattern of scattered light from the particles and hydrodynamics, likely contributing to the coordinated collective motion of the swarm.

## Conclusions

We demonstrated the formation of optical matter inside and outside the irradiation area of a focused laser beam using hybrid metallic-dielectric core–shell nanoparticles with a 200 nm gold core and silica shells of varying thicknesses (0, 22, 50, and 100 nm). Our findings reveal that the silica shell significantly influences the essential properties of optical binding, such as interparticle distance, reducing it below the anticipated optical binding length and the optical matter arrangement. With a thin SiO_2_-shell, optical matter behaves mostly like pure gold, exhibiting an orientation perpendicular to the polarization and dumbbell-shaped assembly at large numbers of particles. For silica shells thicker than 50 nm, we observed a transition from a linear arrangement perpendicular to polarization to a hexagonal arrangement accompanied by a circular motion.

Dynamic numerical calculations corroborated the experimental results, accurately reproducing both the arrangement and motion of the optical matter structures. We also found that the silica shell’s effect can be mitigated by refractive index matching with the solvent, restoring the dumbbell-shaped assembly. However, the optical matter within the irradiated area still exhibited a hexagonal shape, suggesting that electronic repulsion may be a significant factor, as supported by numerical calculations.

Our study highlights the potential of hybrid dielectric–metallic materials in creating optical matter with diverse shapes, arrangements, and dynamics. These findings emphasize the value of hybrid materials in generating different optical matter structures and provide a theoretical framework for the rational design of functional optical matter. This design is crucial for creating materials with desirable optical properties, as has been demonstrated for metamaterials with high or negative refractive indices.^38−42^ The relationship between optical matter structure and their potential properties still needs to be unraveled and will be the topic of future research. We propose that optical matter, a type of nonequilibrium (active) colloidal self-assembly, presents the advantage of being dynamically tunable by changing the light properties with a fast response (<1 s).

## Materials and Methods

### Optical Trapping Setup

The optical trapping setup used is similar to the one described in previous works (Figure S13). Briefly, A 1064 nm continuous wave laser (Spectra-Physics) was guided to the sample and collimated using a 4× beam expander that ensured the filling of the back aperture of the objective. Then, the laser beam is tightly focused approximately 1–2 μm inside the upper glass/solution interface by an air-immersion objective lens (NA 0.90, 60×, Olympus UPlanFLN 60X). The laser power after the objective lens is set to 20 mW. The trapping laser power was controlled by using a half-wavelength plate combined with a polarizer (Thorlabs, VA5–1064). The diameter of the laser is about 1.5 μm at the focal plane (Figure S12). A zero-order half-wave plate (Thorlabs, WPH10M-1064) is used for rotating the direction of linear laser polarization. A halogen lamp illuminates the sample through an oil-immersion dark-field condenser lens (NA 1.2–1.4; Olympus). The scattered light by the trapped AuNPs is collected by the objective, filtered by a short-pass optical filter (Semrock, FF01–1010/SP-25) to remove the 1064 nm laser backscattered light, and recorded using a scientific complementary metal–oxide–semiconductor (sCMOS) camera (Hamamatsu, OrcaFlash) with an acquisition rate of 100 fps.

### Single-Particle Tracking (SPT) Analysis

To track the motion of single particles, we used an in-house written algorithm. The details of the algorithm used for detecting and tracking the NPs are the same as the one used in our previous work.^31^ The code used in this work for tracking is mostly based on the code described in our previous publication.^62^ However, in this case, we took advantage of the fact that we knew exactly how many particles are trapped in each system. Therefore, we made a forced detection of n number of particles by detecting the brightest pixel in the image followed by a deletion of said pixel and the ones surrounding it. We repeated these steps until n particles were detected. After that, Gaussians were fitted simultaneously to the image to optimize the accurate localization of the particle positions. Finally, we compared subsequent frames while minimizing the sum squared displacements to perform the tracking.^62,63^ The algorithm is available at https://github.com/BorisLouis/goldTracking.

### Sample Preparation

The sample was prepared by sandwiching 10 μL of a hybrid Au NP (bAu, AuSS_22_, AuSS_50_, AuSS_100_) colloidal suspension (see synthesis details in the dedicated section) between two clean coverslips with a 120 μm depth spacer (Electron Microscopy Sciences). Before sample preparation, the Au NP suspension was sonicated for 10 min to disperse the NPs. The coverslips were cleaned using an ozone treatment (60 min) to avoid adhesion of the Au NPs to the glass substrate.

### Nanoparticle Synthesis

Chemicals such as gold(III) chloride trihydrate (HAuCl_4_·3H_2_O, ≥99%), sodium borohydride (NaBH_4_, 99%), hexadecyltrimethylammonium bromide (CTAB, ≥99%), l-ascorbic acid (AA, ≥99%), benzyldimethylhexadecylammonium chloride (BDAC), tetraethyl orthosilicate (TEOS, 98%), and sodium hydroxide (NaOH, 97%) were purchased from Sigma-Aldrich–Merck. Ethanol (99.9%) was purchased from Scharlau. All chemicals were used without further purification. Milli-Q water (resistivity of 18.2 MΩ·cm at 25 °C) was used in all experiments. All glassware was cleaned with aqua regia, rinsed with Milli-Q water, and dried before use.

### Synthesis of 200 nm Gold Nanospheres^64^

200 nm gold nanospheres were synthesized via successive seed-mediated growth with controlled diameters increasingly from 10 to 200 nm. First, gold seeds (∼1.5 nm) were prepared by sodium borohydride (0.3 mL, 10 mM) reduction of HAuCl_4_ (0.25 mM, 5 mL) in aqueous CTAB solution (100 mM). After 30 min, an aliquot of seed solution (0.6 mL) was added to a growth solution (100 mL) containing CTAC (100 mM), HAuCl_4_ (0.18 mM), and AA (0.36 mM). The mixture was left undisturbed for 2 h at 25 °C. Upon synthesis, the solution containing 10 nm gold nanospheres was centrifuged (9000 rpm, 2 h) to remove excess CTAC and ascorbic acid and redispersed in an aqueous BDAC solution (15 mM) to a final gold concentration equal to 1 mM.

To grow 10 nm gold nanospheres up to 80 nm, a 10 nm gold nanoparticle solution (24.5 μL, 1 mM) was added under vigorous stirring to a growth solution containing BDAC (50 mL, 15 mM), HAuCl_4_ (0.25 mL, 50 mM), and AA (0.25 mL, 100 mM). The mixture was gently stirred for 30 min at 30 °C and then washed twice by centrifugation (4000 rpm, 20 min). The particles were dispersed in BDAC (15 mM) to a final gold concentration of 1 mM.

To grow 80 nm gold nanospheres up to 200 nm, an 80 nm gold nanoparticle solution (0.85 mL, 1 mM) was added under vigorous stirring to a solution containing BDAC (50 mL, 15 mM) and AA (0.25 mL, 100 mM), followed by dropwise addition of aqueous HAuCl_4_ solution (1.25 mL, 10 mM) using a syringe pump at an injection rate of 1 mL h^–1^. The reaction was allowed to proceed at 30 °C for 30 min after the injection had been finished. The as-synthesized 200 nm gold nanospheres were washed through centrifugation (2000 rpm, 20 min) and redispersed in a CTAB solution (2 mM) to a final gold concentration of 1 mM. The final diameter of gold nanospheres was 200 ± 1 nm. ζ-potential bAu NPs = −28 mV.

Mesoporous silica coating of 200 nm gold nanospheres was carried out by adding aliquots of TEOS solution (each aliquot: 60 μL, 20 vol % in ethanol) at 60 min intervals to a solution containing gold nanospheres solution (5 mL, 1 mM) and NaOH (0.05 mL, 100 mM) in CTAB (2 mM). The reaction was allowed to continue in a water bath at 45 °C while stirring for 1 day, resulting in a silica shell thickness of 22 ± 1, 48 ± 1, and 103 ± 1 nm, for 1, 4, and 8 aliquot additions of TEOS solution, respectively. The resulting silica-coated nanoparticles were centrifuged several times (1500 rpm, 20 min) to remove excess reagents and finally redispersed in ethanol. ζ-potential AuSS_22_ NPs = −31 mV; ζ-potential AuSS_50_ NPs = −25 mV; and ζ-potential AuSS_100_ NPs = −27 mV.

### Numerical Calculations

The dipolar electric nature of the nanoparticles has been guaranteed by means of Mie scattering calculations, which provide complex-valued polarizability (α_e_) through the corresponding Mie coefficient. If *E*(*r*_*i*_,*t*) stands for a phasor representing the total electric field at a dipole located at *r*_*i*_ in time *t*, then the force on the electric dipole is given by^65^
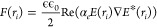
1where ϵ is the dielectric constant of the surrounding media. The electric field results from the superposition of the incident beam and the scattering of all other particles. If we represent the Green’s function propagator with *G*(*r*_*i*_,*r*_*j*_) then the total electric field on a dipolar particle at position *r*_*i*_ equals^66^
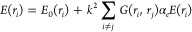
2where *E*_0_(*r*) stands for the external electric field, the wavenumber *k* = 2π*n*/λ_0_ considers the index of refraction *n* = √ε of the medium, and the sum extends over the positions of all other suspended nanoparticles.

To calculate the optical force on the nanoparticles, we first need an accurate model for the incident beam, a laser focused on a diffraction-limited spot close to the water–glass interface. To this end, we follow the Debye–Wolf decomposition and divide up the available incident directions (determined by the experimental numerical aperture) into a discrete set of wave vectors *k*_*n*_. The external field is then represented by the following superposition of plane waves:
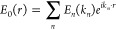
If θ and ϕ represent the polar angles, the corresponding wave vectors are given by *k*(sin(θ)cos(ϕ), sin(θ)sin(ϕ), cos(θ)) while the complex-valued amplitudes are obtained from
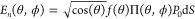
with  being the apodization factor and *f*(θ) being the Gaussian beam profile of the laser and *P*_0_ the polarization axis (we consider a *y*-polarized beam with *P*_0_ = (0, 1, 0)). Orthogonality is imposed by considering the following rotation matrix:
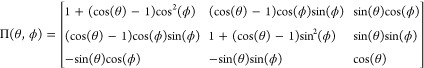
Instead of a direct discretization of the polar angles, we used a Fibonacci lattice on the spherical cap to obtain a more homogeneous distribution of wave vectors. Therefore, we were able to use a constant area element d*S* equal to the surface of the spherical cap divided by the number of propagation directions considered. Reflectance on the water–glass interface remains below 1% and is not considered. Also, due to the low refractive index contrast between the water–glass interface, dipole image corrections are not considered. For all of the simulations discussed below, the focus position is set to *x* = *y* = 0, *z* = 1000 nm above the water–glass interface, and a numerical aperture given by *n* sin(α) of NA = 0.9 is considered. The nominal laser power is estimated by matching the simulated position histogram of a single trapped nanoparticle with the one observed in the experiments (SI Note I, Figure 1 teo).

Once the illumination field is established, we must solve the scattered fields on each particle using the equation (eq 2). Usually, in discrete dipole approximations, this is done by computing the matrix inversion. However, due to the large number of calculations required in the molecular dynamics simulations, we found it more practical to calculate the field by successive iterations, using *E*_0_(*r*_*i*_) as an initial approximation for the total field at time *t* (*E*(*r*_*i*_,*t*)) and then repeatedly substituting the solution into equation (eq 2) until convergence is obtained. With the total fields resulting from this iterative process, we obtain the optical force on each particle using the equation (eq 1).

To explicitly consider the fluid velocity fields affecting the nanoparticles, the dynamics must deal with both Brownian motion and hydrodynamic effects.^67,68^ To simulate these two processes in time, we use Brownian Dynamics^69^ (overdamped Langevin equations of motion) with hydrodynamic interactions, embodied in the following stochastic differential equation.^70^
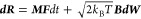
In this equation, *dR* represents the displacement of the particles, **M** is the Rotne–Prager–Yamakawa mobility matrix,^71,72^***F*** is the total force on each particle, *k*_B_ is Boltzmann’s constant, *T* is the absolute temperature and ***dW*** is the Wiener process. To ensure the fulfillment of the fluctuation–dissipation theorem, matrix **B** is determined from the condition *BB*^*T*^ = *M.*(69,73) This hybrid hydrodynamic-optical approach to analyze the dynamics of nanoparticles has already been successfully applied to optical lattice systems.^67,68^

In addition to the optical forces, ***F*** includes short-range interactions among particles and between particles and the glass interface. These are described with the Weeks–Chandler–Andersen repulsive potential.^74^ Also, colloidal particles are charged, so we expect a repulsion force at short distances, in accordance with the Gouy–Chapman theory. We mimic this repulsion by introducing an *ad hoc* exclusion distance larger than the hydrodynamic radius. Setting this exclusion distance to a value of 100 nm from the nanoparticle surface ensures the fulfillment of the dipole approximation in our calculations.

## Supporting Information Available

A schematic of the simulated system used in numerical calculations, additional statistics for two-particle cases, numerical calculation of the optical force as a function of the silica shell thickness, correlation of motion in two-particle system in Cartesian vs polar coordinates, exemplary data for the three-particle system case for all silica shell thicknesses, a schematic of rotation angle used in Figure 3, examples of different optical matter structures obtained with AuSS_50_, a demonstration of the rotation of AuSS_100_ via rotation of linear polarization’s direction, effect of electric double layer on binding distance, a plot of dielectric to metallic ratio in volume and in surface for the different silica thicknesses, a figure on the torque around the geometric center for 3 particles cases of the different silica shell thickness, a measurement for the trapping laser beam size from reflection of the interface, and a schematic of the experimental setup, and supplementary bibliography (PDF)
