# Supplementary material for: Unconventional Optical Matter of Hybrid Metal–Dielectric Nanoparticles at Interfaces
Source: ACS Nano. 2024 Nov 18;18(47):32746–58. doi: 10.1021/acsnano.4c10418 (PMC11614098; doi:10.1021/acsnano.4c10418)
Supplement: Supplementary file 1 — nn4c10418_si_001.pdf [file nn4c10418_si_001.pdf]

Unconventional optical matter of hybrid metal-dielectric nanoparticles at interfaces

Boris Louis,[1,+] Chih-Hao Huang,[2,+] Marc Melendez,[3,+] Ana Sánchez-Iglesias,[4,5] Jorge Olmos-Trigo,[6] Sudipta Seth,[1] Susana Rocha,[1] Rafael Delgado-Buscalioni,[3] Luis M. Liz-Marzán,[4,7,8] Manuel I Marqués,[3] Hiroshi Masuhara,[2,*] Johan Hofkens,[1,9,*] Roger Bresolí-Obach[1,10,*]

1 Laboratory for Photochemistry and Spectroscopy, Division for Molecular Imaging and Photonics, Department of Chemistry, Katholieke Universiteit Leuven, Leuven 3000, Belgium.
2 Department of Applied Chemistry and Center for Emergent Functional Matter Science, National Yang Ming Chiao Tung University, Hsinchu 300093, Taiwan.
3 Departamento de Física Teorica de la Materia Condensada & Condensed Matter Physics Center (IFIMAC), Universidad Autónoma de Madrid, C. Francisco Tomás y Valiente, 7, 28049 Madrid, Spain.
4 CIC biomaGUNE, Basque Research and Technology Alliance (BRTA), 20014 Donostia-San Sebastián, Spain
5 Center for Materials Physics (CSIC-UPV), 20018 Donostia-San Sebastián, Spain
6 Departamento de Física, Universidad de La Laguna, Apdo. 456. E-38200, San Cristóbal de La Laguna, Santa Cruz de Tenerife, Spain.
7 Ikerbasque, Basque Foundation for Science, 48009 Bilbao, Spain
8 CINBIO, Universidade de Vigo, Departamento de Química Física, Campus Universitario As Lagoas, 36310 Marcosende Vigo, Spain
9 Max Planck Institute for Polymer Research, Mainz 55128, Germany.
10 AppLightChem, Institut Químic de Sarrià, Universitat Ramon Llull, Barcelona 08017, Spain.
+ these authors contributed equally.

Contents

Numerical calculations – Schematic of the simulated system 2
Two particle-systems statistics 3
Optical force vs silica shell thickness 4
Two particle-system motion correlation: cartesian vs. polar coordinates 5
Three particle-system case 6
Rotation angle schematic 7
$AuSS_{50}$ photo-stationary optical matters 8
Rotation of assembly with light polarization 9
Electric double layer 10
Plasmonic-dielectric ratio 11
Restoring force 12
Laser focus beam size 13
Optical Setup 14

Numerical calculations – Schematic of the simulated system

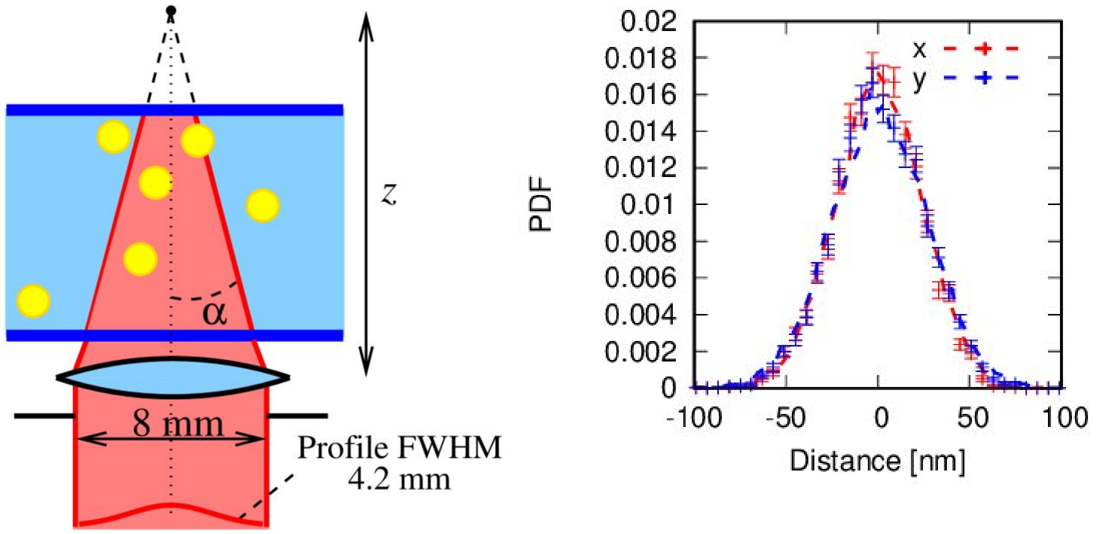

Figure S1: ***Left***: **schematic representation of the system analysed in the simulations. The laser beam impinges at the objective aperture with an intensity profile that approximates a Gaussian distribution with full width at half maximum (FWHM) of 4.2 mm. The laser is then focused on a point located approximately 1 micrometre above the top water-glass interface.** ***Right***: **Probability density function on the *x* and *y* coordinates for a particle trapped in an optical tweezer. The points represent the experimental data and the dashed line the simulation results after force intensity calibration. Numerical aperture** $NA = n\ sin\alpha = 0.9$**.**

Two particle-systems statistics

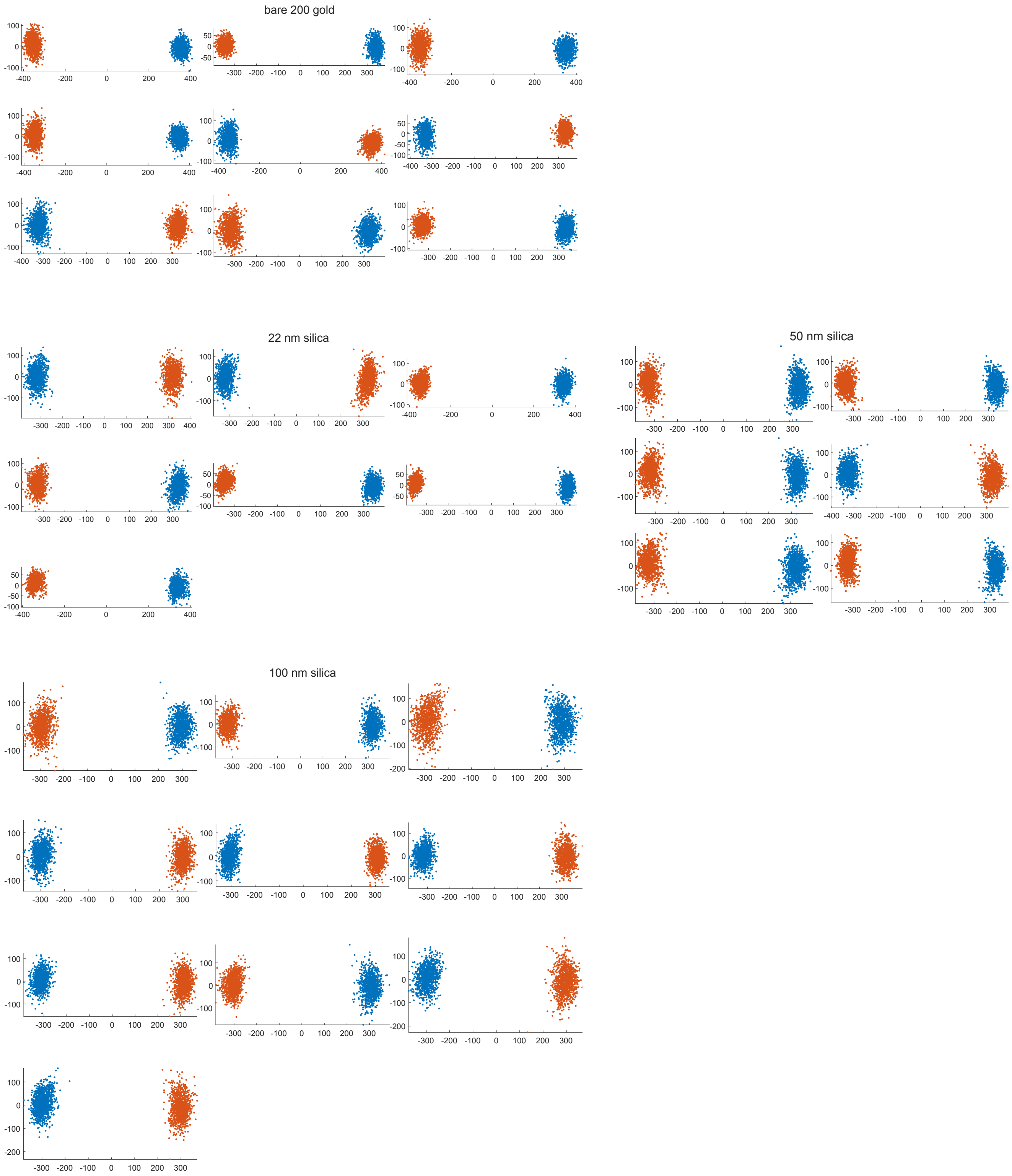

Figure S2: Additional example of localization data for 2 particle case for the different silica shell thickness samples bAu, $AuSS_{22}$, $AuSS_{50}$, $AuSS_{100}$.

Optical force vs silica shell thickness

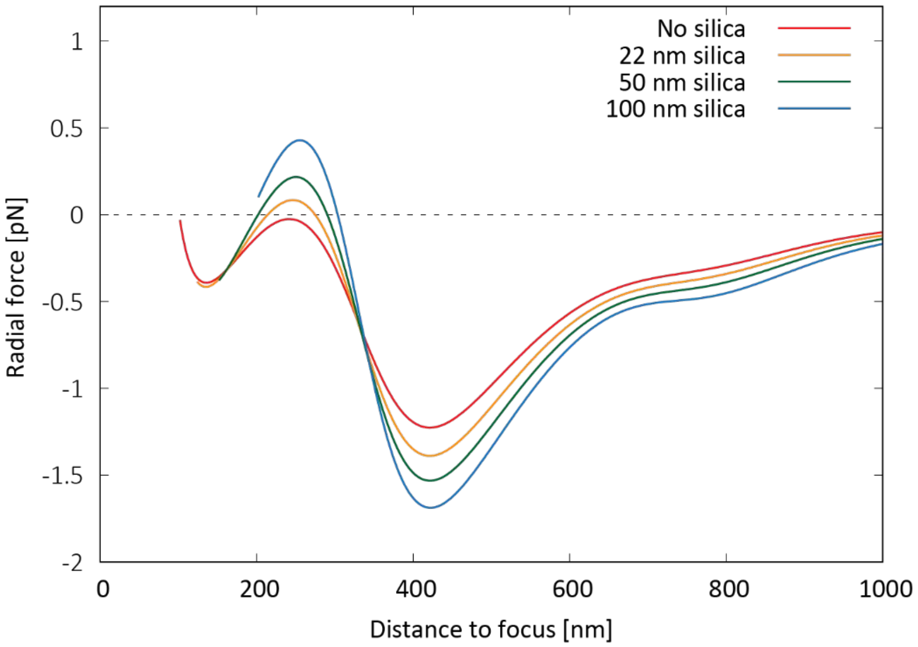

Figure S3: Optical binding force experienced by two particles symmetrically placed on the *x* axis around the focus of an optical trap versus the distance to focus. Several silica shell thicknesses are considered. The intercepts with the *x* axis indicate points of equilibrium. Those with positive slopes are unstable, while negative slopes correspond to stable points. At the stable equilibrium points, greater slopes indicate stronger binding.

Two particle-system motion correlation: cartesian vs. polar coordinates

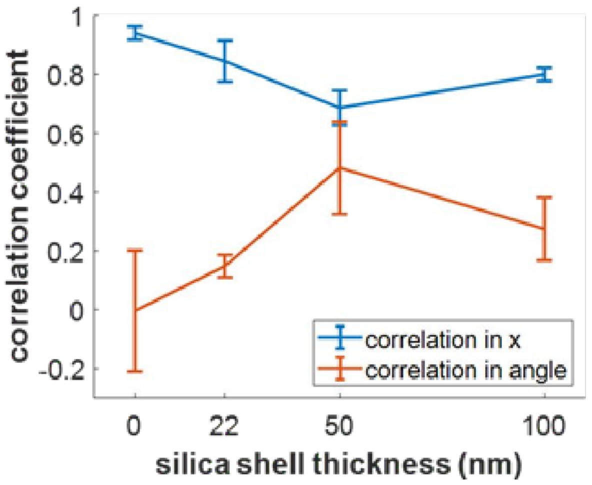

Figure S4 Correlation coefficient: Blue solid line represents the correlation in the x axis, corresponding to the direction perpendicular to the polarization and optical binding direction. Orange solid line represent the correlation in angle, indicating that a slightly rotative oscillation appears as the silica shell increases.

We note here that the correlation in x axis (perpendicular to the polarization) is slightly inversely proportional to the correlation in angle. This can be easily understood as the slight rotation movement may tend to give a slight anti-correlation in the x and y axis which yield lower correlation compared to bare gold where no rotation is observed.

Three particle-system case

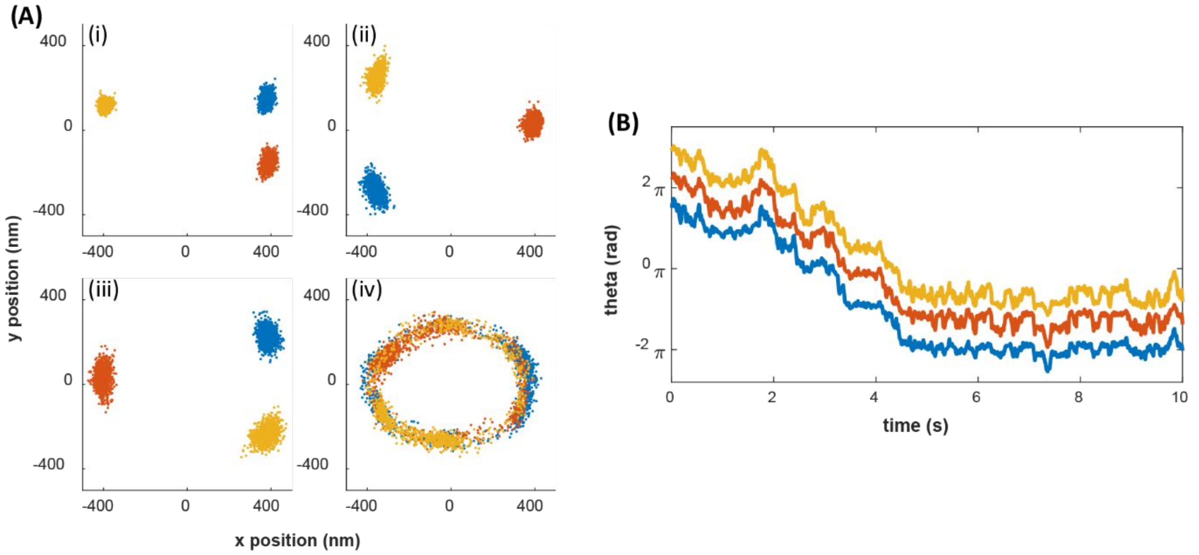

Figure S5: Experimental data for 3 particle case. A) Localization map for different silica shell thicknesses (i) bAu, (ii) $AuSS_{22}$, (iii) $AuSS_{50}$, (iv) $AuSS_{100}$ – B) Exemplary tracked traces for 100nm silica shell after polar coordinate transformation, showing rotational behavior with regular change of direction. Note that even though the trend shown here seems to be majorly along one rotational direction, the rotational motion is mostly a-directional with regular change of direction and henceforth, the time-average angular momentum is likely 0.

Rotation angle schematic

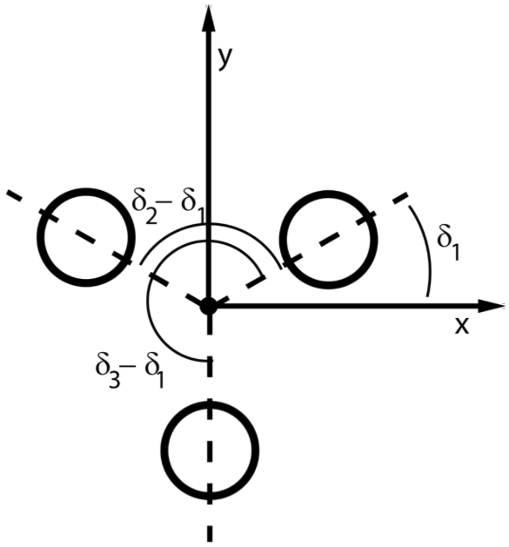

Figure S6: Rotation angles with respect to the center of mass and *x* direction, for three trapped particles, as used in Figure 3.

AuSS$_{50}$ photo-stationary optical matters

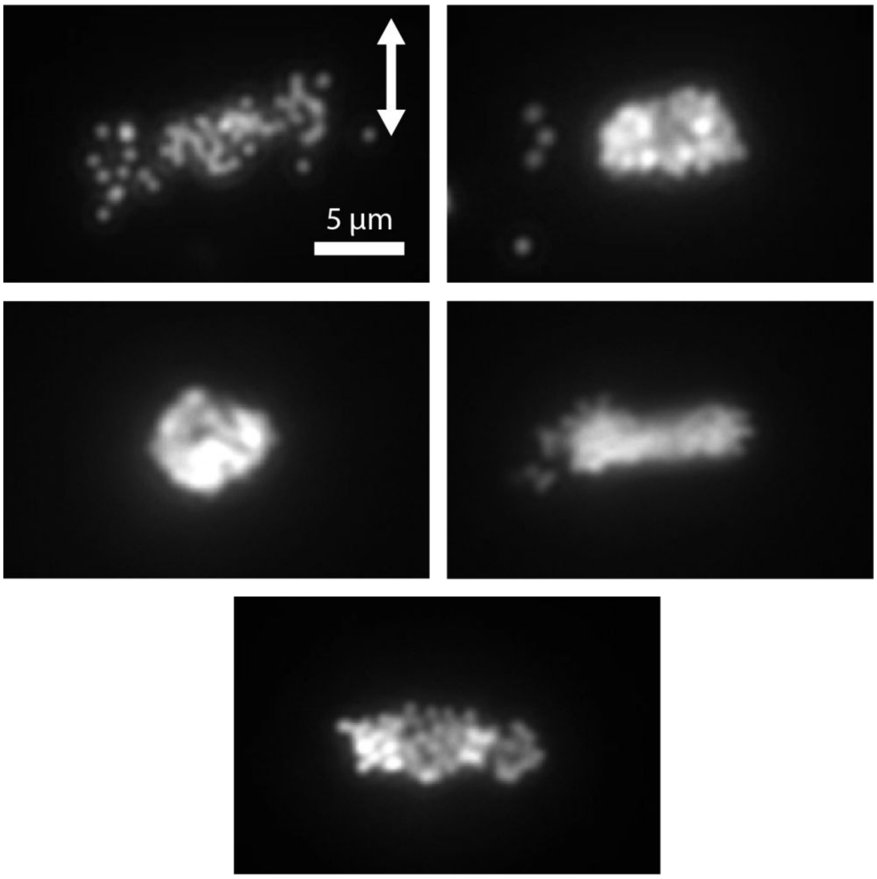

Figure S7 Au50SS assembly shape: While mostly presenting a shape similar to the dumbbell with orientation perpendicular to the light polarization, these particles also present different shapes, and also dumbbell shaped assembly with reduced interparticle distance and consequently reduced dynamic.

Rotation of assembly with light polarization

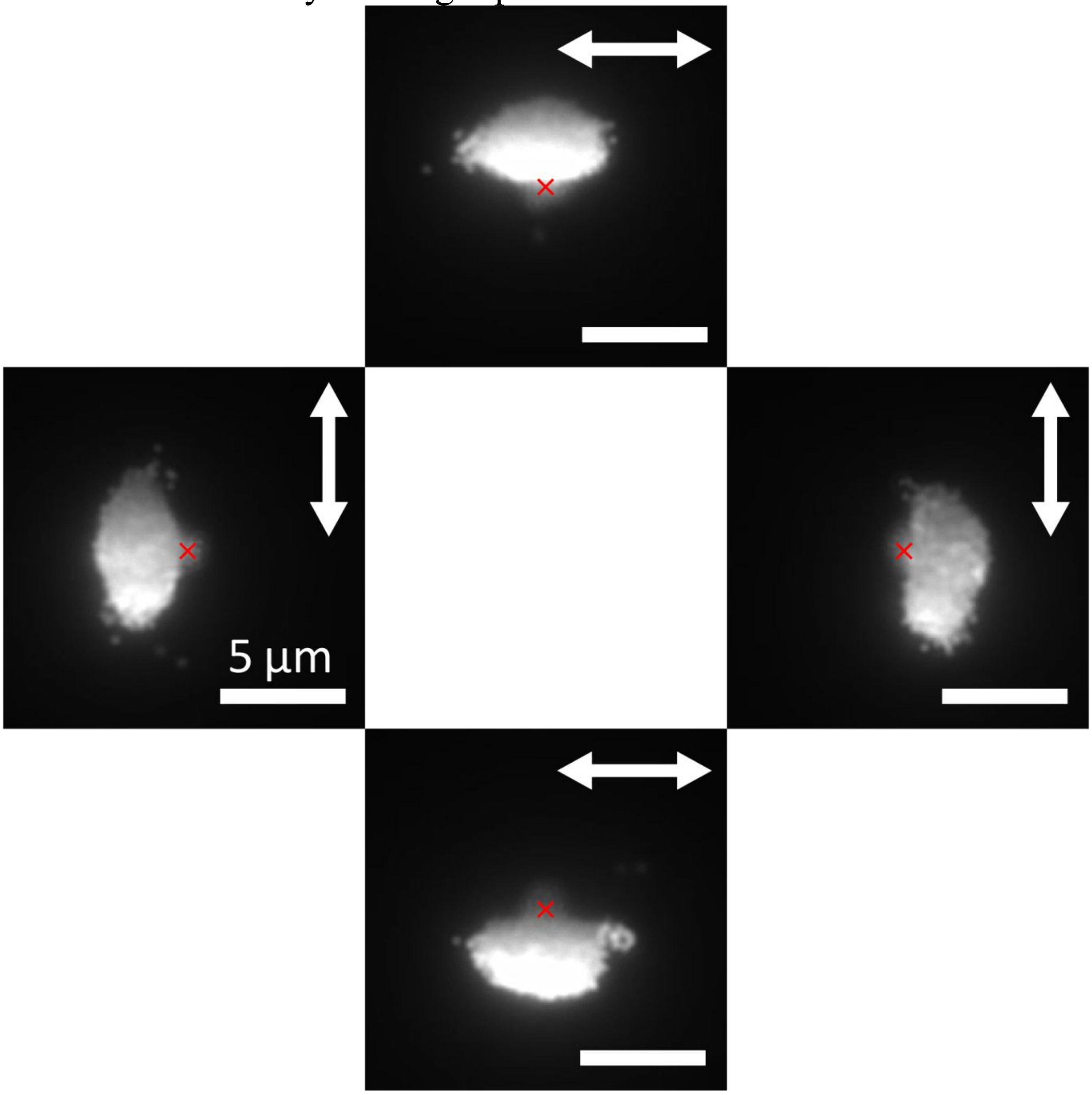

Figure S8 Assembly rotation using light polarization showing AuSS100 assembly for different polarization orientation.

Electric double layer

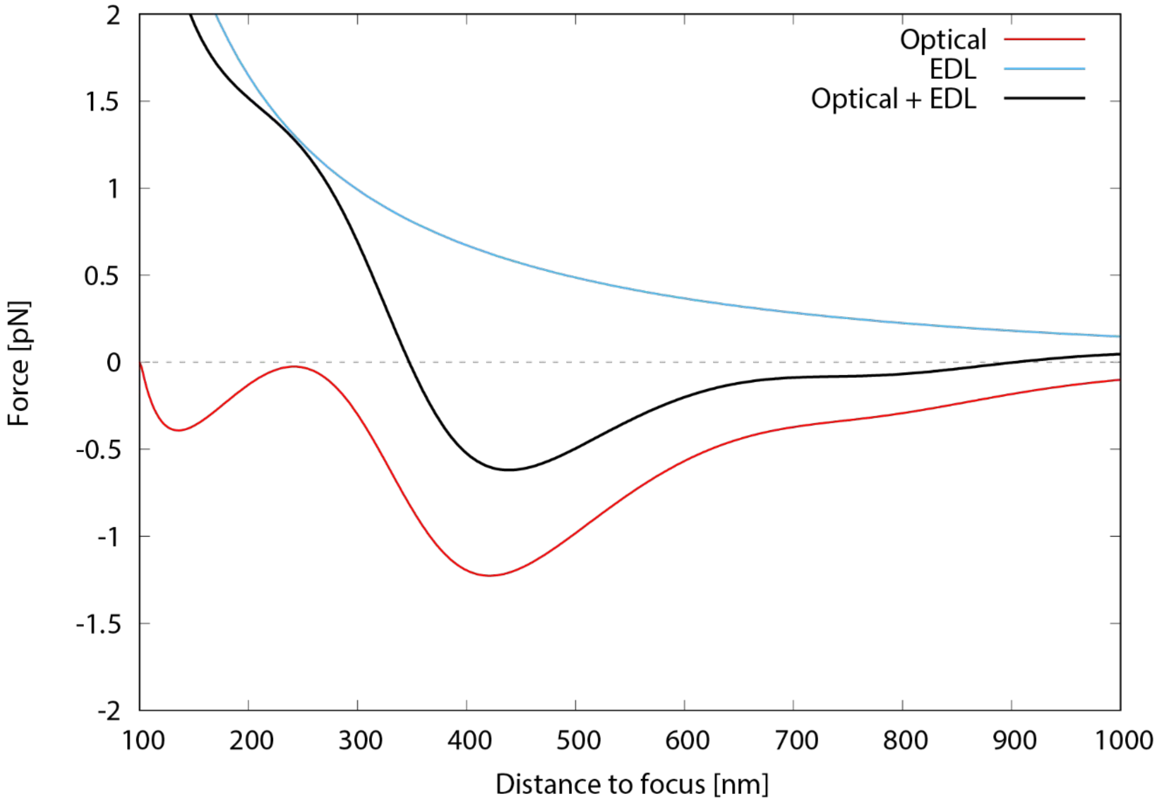

Figure S9: Total binding force (in black) versus distance to focus experienced by two 200-nm gold spheres due to the combined effect of the repulsive electrostatic double layer (EDL, *in blue*) and the optical forces (*in red*). A stable point at roughly 350 nm from the focus emerges when EDL interactions are considered (κ=10^{6} m^{-1}).

Plasmonic-dielectric ratio

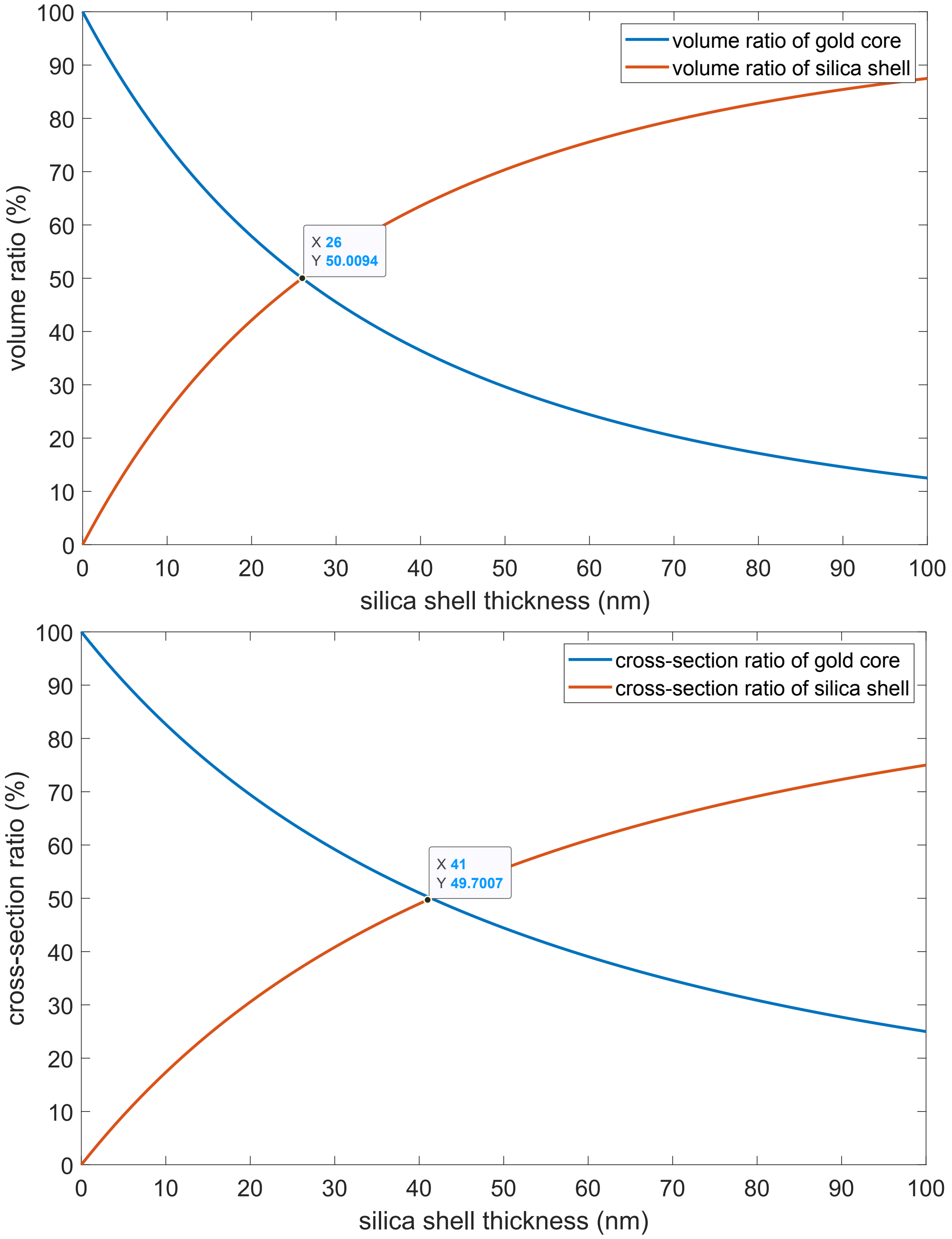

Figure S10 – Dielectric to plasmonic ratio. Top: Volume ratio Silica (dielectric) to gold (plasmonic) Bottom: Surface ratio Silica (dielectric) to gold (plasmonic).

Restoring force

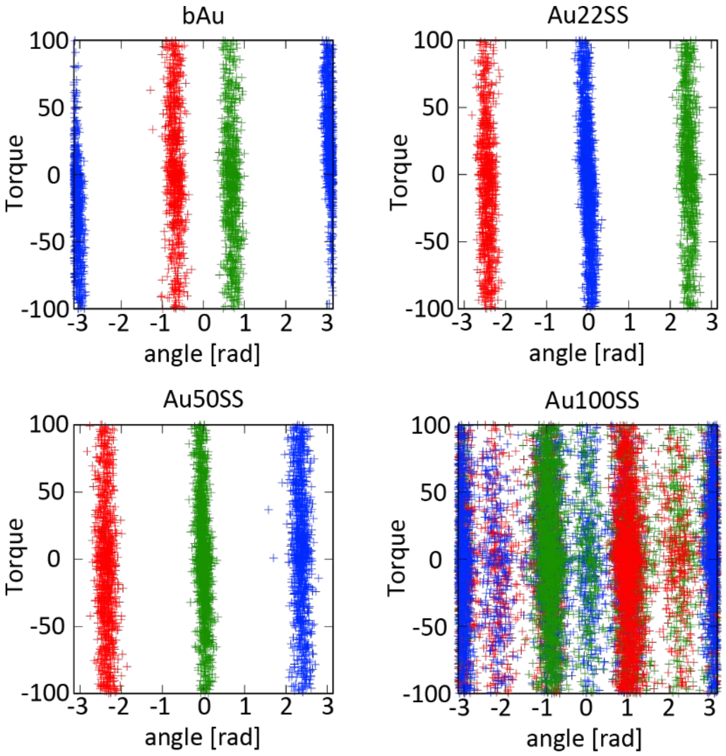

Figure S11. Torque around the geometric center (in simulation units) felt by three trapped spheres in different configurations as a function of the angles δ_i (see Fig. S6) for four different silica coverings. Each colour represents a different particle. A very large restoring force is observed for deviations from the equilibrium configuration, except for the 100-nm-thick silica shell, where very small torques are sometimes observed in between stable mirror configurations.

Laser focus beam size

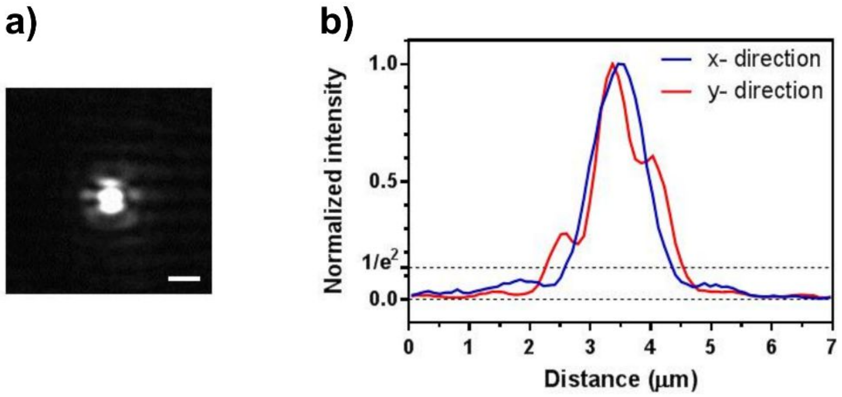

Figure S12. Experimental determination of the laser beam size. a) back-reflection image from the trapping laser at the optical conditions used in this work. b) Normalized laser intensity profile along the x- and y- directions. The scale bar is 2 mm, and the horizontal dashed line indicates the normalized intensity value of $1/e^2$.

However, the trapping laser profile using the back-reflection images is convoluted with the microscope system's point spread function and can be modified to some extent by its partial refraction at the interface and the dichroic mirror. Thereby, we defined the focal diameter in Main text (such as shadowed grey circle in Fig. 2, 3 and 4) as 1.8 mm which is theoretically derived.

Optical Setup

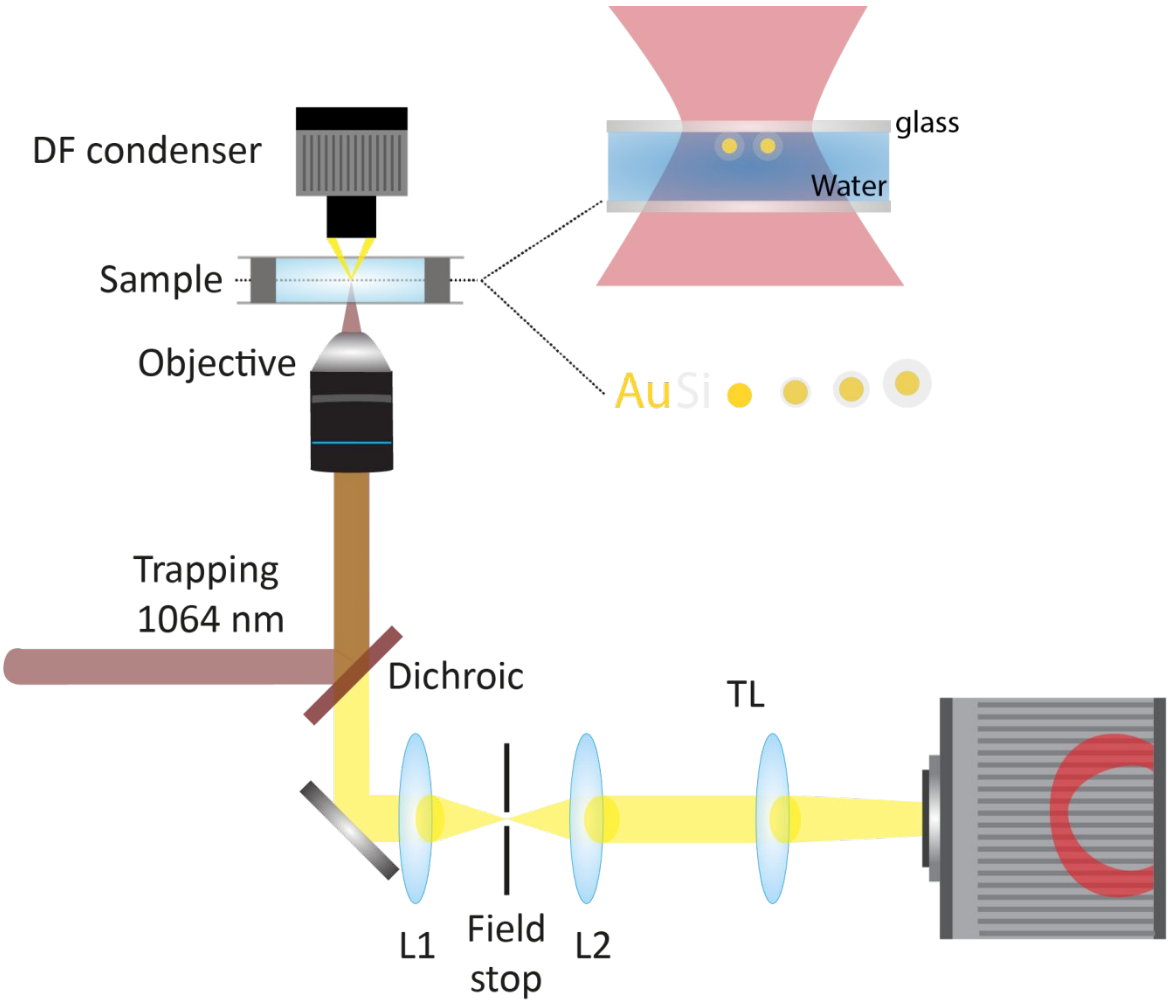

Figure S13 Optical trapping setup: 1064nm Nd:YAG laser is focused at water-glass interface, creating the unconventional optical matter structure. A darkfield condenser enables the observation of forward scattering by the nanoparticles, which is collected by the objective and imaged on the camera after passing through an 8f lens system.
